# Patient-centered evaluation of alignment changes during the first stage of orthodontic treatment: A longitudinal observational study

**DOI:** 10.15171/joddd.2019.012

**Published:** 2019-04-24

**Authors:** Aydin Sohrabi, Shabnam Tahamtan, Ali Rafighi, Seyed Hossein Moslemzadeh, Sana Seyedshariatdoost

**Affiliations:** ^1^Department of Orthodontics, Tabriz University of Medical Sciences, Tabriz, Iran; ^2^Department of Orthodontics, Isfahan University of Medical Sciences, Isfahan, Iran; ^3^Dentistry Student, Faculty of Dentistry, Tabriz University of Medical Sciences, Iran

**Keywords:** Alignment, orthodontic treatment, tooth alignment

## Abstract

***Background***. The aim of this longitudinal observational study was to evaluate patients’ perceptions of alignment changes during the first stage of fixed orthodontic treatment.

***Methods***. Ninety-three non-extraction patients (mean age: 17.6 years) who were scheduled to undergo fixed-appliance treatment in the first author's private office were included. Patients assessed the alignment of their teeth subjectively using visual analogue scale at the bonding session and four, eight and 12 weeks later. The amount of Little's irregularity index at each session was calculated on stone casts. Freidman test was used to compare the "alignment changes" between different intervals. Correlation coefficients were calculated using Spearman test between Little’s irregularity indices and alignment scores reported by the patients in each session.

***Results***. No patients reported regression in alignment changes during three-month course of treatment. The final changes (from bonding session to the 12th-week visit) were smaller than the sum of the three intervals, which indicated that patients became more perceptive as the treatment progressed. Comparison of two scores reported for each session (in the same session and in the next session) revealed that patients could not recall their previous situation well. Patients do not perceive alignment changes in the same way as clinicians. Furthermore, patients who were 16 or older perceived smaller alignment changes during the first four-week period and smaller final alignment changes.

***Conclusion***. To obtain better patient compliance and improve their motivation throughout orthodontic treatment, patientspecific measures should be undertaken, including reminding them about their initial conditions and highlighting the changes as the treatment progresses.

## Introduction

Currently, oral health is defined as “a comfortable and functional dentition, which allows individuals to continue in their desired social role.”^1^ Accounting for social and quality of life aspects, it is essential to evaluate the patient's perspectives.^2^ Such perceptions can have great importance in making delivery of orthodontic care more satisfying for both patients and orthodontists.^3-8^ It is known that patients’ perspectives do not necessarily bear resemblance to clinical measures.^9^

Patients’ expectations affect their evaluation of the quality of treatment and their satisfaction with treatment outcomes.^10^ They evaluate treatment efficacy by comparing their expectations with the real outcomes.^11^ If a patient's expectations are not met, it might cause dissatisfaction, resulting in failure to achieve professional success.^11,12^

Although orthodontic treatment is primarily aimed to benefit the patient, most of the studies overlook patient concerns when evaluating the treatment. Recently, Tsichlaki and O’Brien assessed how orthodontic research outcomes reflect patient values, reporting that 63% of the RCTs in the field of orthodontics have focused on professional outcomes, with little emphasis on patient’s perspectives,^2^ and the reported outcomes are mostly relevant to clinicians and are not patient-centered.^13,14^

Although there is emphasis on patient-centered evaluation of treatment outcomes, most studies that have evaluated patients’ attitudes toward and perceptions about treatment results have evaluated those outcomes only at the end of treatment. Fixed orthodontic treatment is accomplished in three stages and completed in 18 to 24 months. During the first stage which is the patient’s first experience with orthodontic treatment, the tooth alignment improves and the PAR index decreases.^15^ Patients’ perceptions during this stage might influence their attitudes toward the subsequent stages and their adherence to treatment. The aim of the present study was to evaluate patients’ perceptions of alignment changes during the first stage of orthodontic treatment.

## Methods

This longitudinal observational study was undertaken from June 2016 to February 2018. A pilot study was conducted initially with 12 patients, in which the mean changes in alignment from the bonding session to the 4th-week visit was 1.61 cm measured using VAS (SD=1.87); based on this result, assuming a margin of error of half unit, a 5% alpha significance level, and 80% power, the required sample size was calculated at 54. Considering a drop-out proportion of one-third and to increase the power of the study, 93 patients were included in this investigation. These were non-extraction cases with crowding (11 to 34 years of age [mean: 17.6 years]; 16 males and 79 females), who were scheduled to undergo fixed orthodontic treatment in the first author's private office.

The study population was drawn from patients starting their orthodontic treatment (with 0.022×0.028-in slot size pre-adjusted edgewise appliances). The inclusion criteria for patient selection were as follows: crowded cases in which all the teeth could be engaged (based on clinician's judgment), presence of all the permanent teeth except third molars; no quadhelix or other palatal expansion devices needed; no extraoral appliances; patients 11 years of age or older, with better understanding of alignment to be able to explain their perspectives.

**Figure 1:**
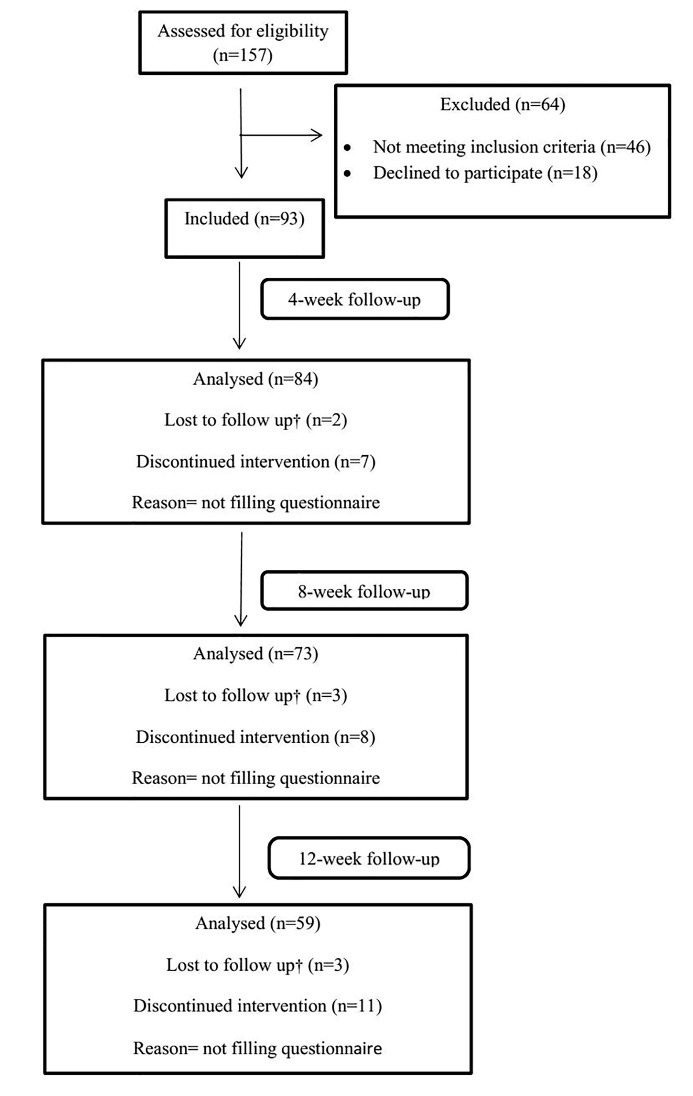
Flow chart of the study design (†missed appointment: has missed or rescheduled his/her appointment for more than 6 days later).

Exclusion criteria consisted of previous orthodontic treatment, considerable medical history or craniofacial abnormality, notable skeletal asymmetry, previous extraction; blocking out of the tooth that could not be bonded at the beginning of treatment; spacing; and unwillingness of the patient to participate in the study. The flow chart diagram detailing patient flow thorough the study is shown in Figure 1.

Subjects who fulfilled the selection criteria were identified and invited to take part in this study. Informed consent was obtained prior to bonding session. On the day of bonding, an office assistant, who specially had been trained for cooperation in this study, asked patients to look at their lower arch teeth in the mirror from front and top views. Then, they were asked “how aligned are your teeth today?” and instructed to mark their alignment on a visual analogue scale (VAS) line. The questionnaires included the horizontal line of the VAS, with “happy” and “sad” pictographs at each end; in addition, written instructions were given regarding how to record their alignment on VAS lines. Four, eight and 12 weeks after the bonding session, the patients received questionnaires with two questions: (1) “How aligned are your teeth today?” and (2) “How aligned were your teeth in the previous visit?” During all the sessions, they were asked to complete the questionnaires while waiting for their appointment.

The alignment change was calculated as follows:

Alignment score in each visit - alignment score in the previous visit

Considering that patients rated the alignment of each session twice (during that visit and during the next visit), for higher representation and greater reliability of how the patients perceived their dental alignment, the numbers given by the patient in the same session were considered to calculate the alignment change between the two sessions.

At the beginning of the study and before placement of brackets, alginate impressions were taken for study models, and irregularity of lower teeth was measured on study models from the right first molar to the left first molar and a modified Little’s irregularity index was calculated for each cast. All the measurements were made by one investigator (S.T), using a digital caliper (at 0.01 mm).

Intraobserver reliability was assessed by performing measurements for Little's irregularity index at a two-week interval on 45 casts with intraclass correlation coefficient test (ICC), which showed excellent agreement between the two measurements (95% confidence interval: 0.97‒0.99).

Impressions were taken again and the irregularity index was re-calculated 4, 8 and 12 weeks later.

### Statistical Analysis

Demographic and patient-opinion data were investigated using conventional descriptive statistics. Comparison of the “alignment changes” between different intervals was undertaken using the Freidman test. To evaluate the reproducibility of the patients when assessing their alignments at different sessions, an agreement test using the intraclass correlation coefficient (ICC) was performed. Correlation coefficients were calculated using Spearman test between Little’s irregularity indices and alignment scores reported by patients in each session. Patients’ perspectives were compared between two age groups of below and above 16 years of age using Mann-Whitney U test. All the analyses were performed with SPSS 21.0, and the level of statistical significance was set to 0.05.

## Results

Although the patients reported the greatest changes during the first 4-week period (mean: 1.81 units), there were no statistically significant differences between the evaluation periods (*χ*^2^= 1.716, *df*= 2, P= 0.424).

Descriptive statistics for the alignment changes reported by patients at different intervals are presented in Table 1.

**Table 1:** Descriptive data of the alignment changes according to the patients’ perceptions

	**Sample size**	**Mean**	**Standard deviation**	**Min- Max**	**P-value**
**Alignment changes during the first interval** ^ 1 ^	84	1.81	1.68	0.0- 7.0	
**Alignment changes during the second interval** ^ 2 ^	73	1.36	1.55	0.0- 5.0	0.424^4^
**Alignment changes during the third interval** ^ 3 ^	59	1.48	1.72	0.0- 9.0	

1. Calculated as “Alignment score at 4th week” *minus* “Alignment score of the bonding session reported in the 4th-week visit” 2. Calculated as “Alignment score at 8th week” *minus* “Alignment score of the 4th week reported in the 8th-week visit” 3. Calculated as “Alignment score at 12th week” *minus* “Alignment score of the 8th week reported in the 12th-week visit” 4. P-value for comparing three intervals using Friedman test

**Figure 2:**
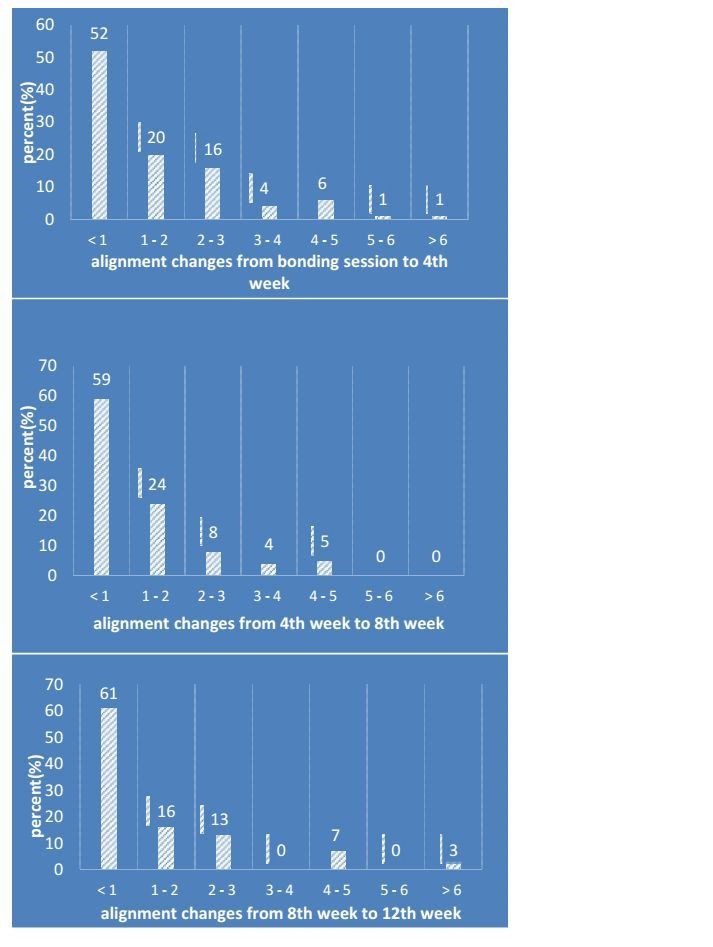
Frequencies of alignment changes as reported by the patients.

Evaluation of the data on alignment changes in the first time interval (from the bonding session to the 4th week) showed that almost half of the patients (48%) mentioned positive changes for tooth alignment (more than one unit [one cm on VAS line]). In the second (4th week to 8th week) and third (8th week to 12thweek) time intervals, 41% and 39% reported this amount of change (more than one unit), respectively (Figure 2). Based on the scores reported by the patients at each interval, it was found that no patients perceived regression of changes for their tooth alignment in the course of the study and the lowest amount of reported change at each interval was zero.

**Figure 3:**
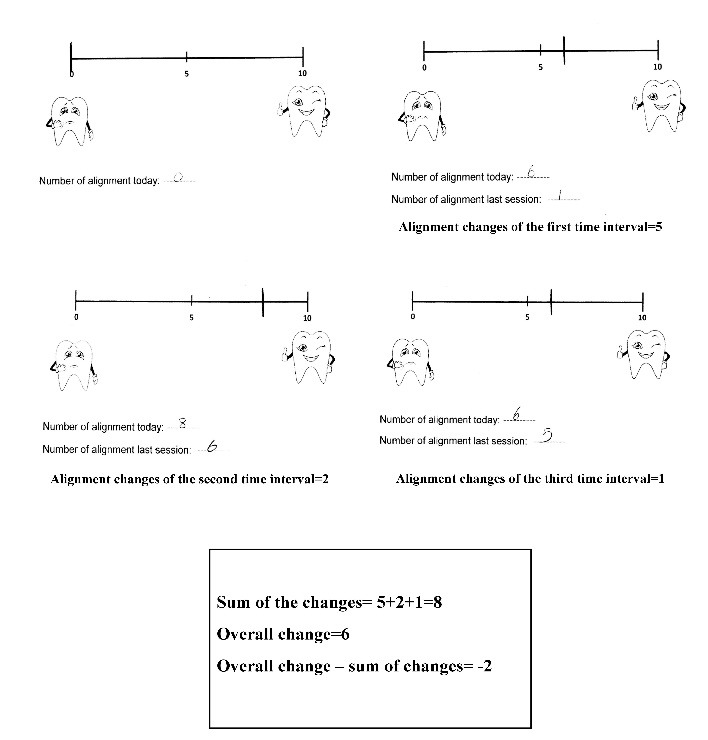
Alignment scores reported by an individual patient (from top left: bonding session, 4th week, 8th week and 12th week).

Alignment scores reported by an individual patient are shown in Figure 3. Comparing the sum of changes for the three intervals and the “final change” (i.e. from the bonding session to the 12th-week visit) showed that these two did not match and even had a poor correlation (Spearman’s ρ=0.39). It was found that although all the patients reported improved alignment at each visit, by the time of the second and third intervals, they became more concerned about the alignment changes, as the mean difference between the final changes (from the bonding session to the 12th-week visit) and the sum of the three intervals was -1.66 cm measured on VAS. Descriptive data comparing final alignment change vs. sum of changes reported by the patients at each visit are shown in Table 2.

**Table 2:** Descriptive data comparing the final alignment change vs. the sum of changes reported by the patients at each visit (n=59)

	**Mean**	**Standard deviation**	**Min-Max**
**Sum of the alignment changes reported by patients in the three intervals** ^ 1 ^	5.13	3.49	0.0 - 17.0
**Final change reported by patients** ^ 2 ^	3.47	2.62	-1.0 - 10.0
**Difference** ^ 3 ^	-1.66	3.30	-7.0 - 5.0

1. Calculated as “Alignment score at 4th-week” *minus* “Alignment score of the bonding session reported in the 4th-week visit”) plus (“Alignment score at 8th week” *minus* “Alignment score of the 4th week reported in the 8th-week visit”) plus (“Alignment score at 12th week” *minus* “Alignment score of the 8th week reported in the 12th-week visit”). 2. Calculated as “Alignment score at 12th week” *minus* “Alignment score of the bonding session reported in the 4th-week visit” Calculated as “Final changes” *minus* “sum of the alignment changes reported by the patients at the three intervals”

At each visit, patients were asked to report the alignment of that day and to report the alignment of the previous session. Following this method, they reported alignment scores for each visit twice (once at the specified session; and second, at the upcoming session). To evaluate how good, the patients recalled their dental alignment, these two corresponding scores were compared by interclass correlation coefficient tests (Table 3), which indicated that the patients remembered the alignment of previous sessions moderately.

**Table 3:** Reproducibility of the self-reported alignment scores assessed using the interclass correlation coefficient (ICC)

	ICC
**Alignment scores of bonding session** ^ 1 ^	.56^**^
**Alignment scores of 4** ^th^ ** week** ^ 2 ^	.58^**^
**Alignment scores of 8** ^th^ ** week** ^ 3 ^	.42^**^

1. Alignment score of the bonding session reported in that session vs. the bonding-session score reported in the 4th week 2. Alignment score of the 4th week reported in that session vs. the 4th-week score reported in the 8th week 3. Alignment score of the 8th week reported in that session vs. the 8th-week score reported at the 12th week

Descriptive statistics of Little’s irregularity indices are presented in Table 4. The greatest improvement in irregularity index was calculated during the first four-week period (mean: 5.68 mm).

**Table 4:** Descriptive data of the Little’s irregularity indices (mm) and irregularity index changes

	**Sample size**	**Mean**	**Standard deviation**	**Min - Max**
**LII** ^ 1 ^ ** at bonding session**	93	12.01	4.62	1.66- 20.55
**LII at 4th week**	84	6.43	3.73	0.47- 16.18
**LII at 8th week**	73	3.91	2.62	0.0- 11.48
**LII at 12th week **	59	2.59	2.58	0.0- 7.81
**LII changes during the first interval** ^ 2 ^	84	5.68	3.51	-0.4- 16.03
**LII changes during the second interval** ^ 3 ^	73	2.48	2.47	0.54- 11.77
**LII changes during the third interval** ^ 4 ^	59	1.23	1.72	-0.9- 6.47
**Final LII changes** ^ 5 ^	59	9.02	4.35	0.84- 17.80

1. Little’s irregularity index 2. “Little’s irregularity index at 4th-week”* minus* “Little’s irregularity index in the bonding session” 3. “Little’s irregularity index at 8th-week” *minus* “Little’s irregularity index at 4th-week” 4. “Little’s irregularity index at 12th-week” *minus* “Little’s irregularity index at 8th-week” 5. “Little’s irregularity index at the 12th-week” *minus* “Little’s irregularity index in the bonding session”

The results of Spearman’s correlation test between alignment scores and Little’s irregularity indices of each session are described in Table 5, indicating that they were not correlated and patients’ perspectives did not match the professional clinical measures during this three-month course of the study.

**Table 5:** Correlation of alignment scores reported by patients in each session and Little’s irregularity indices of study models taken in the same session

**Evaluation session**	**Compared variables**	***Spearman’s*** ** ρ**	
**Initial**	Little’s irregularity index versus alignment score reported by patients	0.050	
**4th week**	Little’s irregularity index versus alignment score reported by patients	0.071	
**8th week**	Little’s irregularity index versus alignment score reported by patients	-0.020	
**12th week**	Little’s irregularity index versus alignment score reported by patients	0.232	

In this study, the perspectives of patients who were <16 years of age (45 patients at the beginning of the study) were compared with patients ≥16 years of age (48 patients) (Table 6). There was no statistically significant difference in initial Little’s irregularity index between the two groups (P=0.286). However, the older group reported significantly higher alignment scores for their initial condition during the bonding session (2.61 cm measured on VAS) but perceived significantly smaller alignment changes during the first four-week period (1.30 units) and smaller final alignment changes (from the bonding session to the 12th-week visit, 2.71 cm measured on VAS).

**Table 6:** Descriptive data comparing perceptions of the patients <16 years of age vs. patients’ ≥16 years of age

	**<16**		**≥16**		**P-value**
	*n*	Mean (SD)^4^	*n*	Mean (SD)	
**Initial LII** ^ 1 ^ **(mm)**	45	12.78 (5.45)	48	11.75 (4.80)	0.286
**Alignment score reported at the bonding session (cm measured on VAS)**	45	1.31 (1.97)	48	2.69 (2.88)	0.019
**Alignment changes reported during the first interval** ^ 2 ^ ** (cm measured on VAS)**	41	2.35 (1.79)	43	1.30 (1.05)	0.003
**Final alignment changes reported by patients** ^ 3 ^ ** (cm measured on VAS)**	28	4.28 (2.17)	31	2.71 (2.77)	0.007

1. Little’s irregularity index 2. “Alignment score at 4th week” *minus* “Alignment score of the bonding session reported in the 4th-week visit” 3. “Alignment score at 12thweek” *minus* “Alignment score of the bonding session reported in the 4th-week visit” 4. SD: Standard deviation

## Discussion

Currently, great emphasis is placed on considering patients’ perspectives on orthodontic treatment outcomes in order to provide better services to the patients and to receive positive feedback from them. Recently, a study assessing how orthodontic research outcomes reflect patient values found that this fact was overlooked in most of the studies and that the evaluation of treatment outcomes was mostly clinician-centered.^13^

In few previous patient-centered studies, patients’ perceptions and their satisfaction were evaluated at the end of orthodontic treatment. Because orthodontic treatment lasts for considerable time, patients’ expectations and attitudes might change during this period. Therefore, evaluating their perceptions during the treatment process can provide the clinician with useful information in order to improve patient satisfaction and compliance.

In this study, we evaluated the alignment changes during the first phase of orthodontic treatment according to patients’ reports.

Significant variations in patients’ reported alignment scores at the bonding session indicated that patients’ understanding of their tooth alignment was different from each other. Also, there was no specific amount of malalignment that encouraged patients to seek treatment.

No patients reported regression of alignment changes during the three-month course of study, and the minimum reported change was zero. However, they reported smaller changes at the second and third time intervals in comparison to the first time interval, which was not statistically significant. It might be due to the fact that they became more concerned about their alignment at the second and third intervals.

Surprisingly, the final change (the 12th-week alignment score minus the bonding session alignment score) was smaller than the sum of the three reported changes at monthly intervals (Table 2) (mean difference: -1.66 cm measured on VAS). Although patients acknowledged the alignment improvement at three intervals of the study and none of them reported regression of changes, as the treatment progressed, they became more sensitive and reported relatively lower alignment scores during subsequent sessions in comparison to the earlier visits. The alignment scores for an individual patient are shown in Figure 3.

In this study, the patients were asked to evaluate their current alignment in each session and to report how their alignment was in the previous session. By following this method, every patient reported two ratings for the same session, once in each session and next in the upcoming visit. Comparison of these two numbers revealed that an individual's perspective on tooth alignment was not consistent throughout the sessions. This inconstancy fluctuated over time, and the greatest reproducibility of malalignment scores was for the 2 ratings of the 4th-week alignment. The reason behind this inconsistency was that they simply forgot “how their teeth were malaligned” in the last session and highly likely this can be attributed to the patients’ becoming progressively more concerned about their alignment changes in subsequent sessions. Lower inconsistency of the 4th-week alignment scores might be explained by the observation that patients were more pleased about alignment improvements during at first time interval and the fact that mind remembers positive memories more easily.^16^ It was shown that patients’ evaluation of their alignment did not correspond with the clinical measures. This fact must be considered during treatment because patients do not perceive alignment changes in the same way as clinicians do. For example, consider a patient with protrusion of upper incisors and a class II canine relationship. The clinician might be happy with initial alignment changes while preparing the dental arch to engage stainless steel wire for anterior retraction. On the other hand, the patient’s perspective might be different and protrusion of upper incisors might be more important for patient than alignment of the teeth. Taking into account the patients’ preferences and perceptions could improve health outcomes for patients.^17^ Also, patient-centered measures could help clinicians and patients make correct decisions.^18^ Therefore it is recommended that initial conditions of the alignment be reminded to the patient and treatment progression be highlighted in each session in order to motivate the patient and achieve better compliance in the course of orthodontic treatment. It is clear that more patient-centered studies are necessary to compare patients who recalled their former conditions with patients who forgot their initial alignment from the aspect of compliance.

Based on our results, patients ≥16 years of age had a different perspective on alignment changes in comparison to younger ones. Although these groups had comparable LIIs at the beginning of treatment, the older group believed that they had more aligned teeth at first and reported smaller changes at the first time interval. Furthermore, they perceived smaller final changes in comparison to the younger group. This finding can be justified by the fact that patients might become more observant and sensitive about their appearance and tooth alignment as they age, and this difference could be considered from the patient management perspective.^19-21^

## Limitations

The limitations of the study were in terms of generalizability as the patients were just from a private clinic and they cannot represent general dental hospitals patients.

In this study, only non-extraction cases were included and all of the teeth should be engaged depending on clinicians' opinions. Furthermore, some of the patients refused to fill the questionnaires and their perspectives toward alignment changes remained unknown.

## Conclusion

- Not all the patients perceived the alignment changes occurring in each period.
- The patients’ perspective on their tooth alignment was not consistent.
- The patients became more concerned about the alignment changes as the treatment progressed.
- It is better to give patients more knowledge about treatment goals and steps at the beginning of treatment; this can result in a consensus between the patient and the orthodontist regarding improvement gains.
- Patients are more observant and sensitive about their appearance and tooth alignment as they become older.
- For higher compliance and motivation of patients throughout the entire course of orthodontic treatment, patient-specific measures should be undertaken, including reminding them about the initial conditions in their treatment and highlighting the changes.

## Conflict of Interests

The authors declare no conflict(s) of interest related to the publication of this work.

## Authors’ Contributions

The study was planned by AS. The literature‏ review was performed by ST, SS, AR and SM. ST and SS performed the measurements and drafted the manuscript. The statistical analyses and interpretation of data were carried out by‏ AS. All the authors critically revised the manuscript for intellectual content. All the authors have read and approved‏ the final manuscript‏.

## Acknowledgments

This research did not receive any specific grant from funding agencies in the public, commercial, or not-for-profit sectors.

## Funding

Not applicable. This research did not receive any specific grant from funding agencies in the public, commercial, or not-for-profit sectors.

## Ethics Approval

Ethical approval for the study was granted by Ethics Committee of Tabriz University of Medical Sciences.
